# Dispersion of γ-Alumina Nano-Sized Spherical Particles in a Calamitic Liquid Crystal. Study and Optimization of the Confinement Effects

**DOI:** 10.3390/ma7031502

**Published:** 2014-02-27

**Authors:** Sergio Diez-Berart, David O. López, Nerea Sebastián, María Rosario de la Fuente, Josep Salud, Beatriz Robles-Hernández, Miguel Ángel Pérez-Jubindo

**Affiliations:** 1 Departamento de Física Aplicada II, Facultad de Ciencia y Tecnología, Universidad del País Vasco UPV-EHU, Apartado 644, Bilbao E-48080, Spain; E-Mails: david.orencio.lopez@upc.edu (D.O.L.); nerea.sebastian@ehu.es (N.S.); rosario.delafuente@ehu.es (M.R.F.); beatriz.robles@ehu.es (B.R.-H.); ma.perezjubindo@ehu.es (M.A.P.-J.); 2 Grup de Propietas Físiques dels Materials (GRPFM), Departament de Física i Enginyeria Nuclear, E.T.S.E.I.B. Universitat Politècnica de Catalunya, Diagonal 647, Barcelona 08028, Spain; E-Mail: josep.salud@upc.edu

**Keywords:** liquid crystals, MDSC calorimetry, dielectric spectroscopy, polydispersive nanoparticles, confinement, γ-alumina

## Abstract

We report an experimental study on confined systems formed by butyloxybenzylidene octylaniline liquid crystal (4O.8) + γ-alumina nanoparticles. The effects of the confinement in the thermal and dielectric properties of the liquid crystal under different densities of nanoparticles is analyzed by means of high resolution Modulated Differential Scanning Calorimetry (MDSC) and broadband dielectric spectroscopy. First, a drastic depression of the N-I and SmA-N transition temperatures is observed with confinement, the more concentration of nanoparticles the deeper this depression is, driving the nematic range closer to the room temperature. An interesting experimental law is found for both transition temperatures. Second, the change in shape of the heat capacity peaks is quantified by means of the full width half maximum (FWHM). Third, the confinement does not noticeably affect the molecular dynamics. Finally, the combination of nanoparticles and the external applied electric field tends to favor the alignment of the molecules in metallic cells. All these results indicate that the confinement of liquid crystals by means of γ-alumina nanoparticles could be optimum for liquid crystal-based electrooptic devices.

The importance of liquid crystals in the field of materials science is enormous, from theoretical and experimental reasons and, also, from the point of view of applications. Liquid crystal research covers many disciplines, from chemistry (design and synthesis), physics (fundamentals, models, characterization…), biology and medicine (many lyotropic liquid crystals are biocompatible and many biomolecules and macromolecules are mesogens), food and soap industries, thermal, mechanical, electronic and optical engineering (thermometers, biomechanic robots, displays, wave guides, lasers…) [1,2], *etc*.

Among all these industrial applications, liquid crystal-based electro-optical devices are largely spread and very well known. The duality of liquid-like fluidity and crystal-like anisotropy makes these materials ideal for such applications. On the one hand, the intrinsic dielectric and optical anisotropies imply a great variety of technical possibilities, as the compounds can be used in the construction of polarisers, filters, modulators, displays, *etc*. On the other hand, the fluidity of mesophases like the nematic (N) or smectic A (SmA) phases, among others, makes the molecules of the liquid crystal likely to orient *ad lib* by the application of small perturbations to the material (in form of electric or magnetic fields, simple chemical surfactants…). Putting these properties of liquid crystals together, the utility of such compounds in this field of technology is pretty obvious.

In order to design any electro-optical device based on liquid crystalline properties, optimizing not just the electrical and optical, but also the thermal properties of the mesogens, is one of the priorities. The importance of the thermal behavior lies in the fact that the device must be designed to work in a particular temperature range, the so called temperature window, depending on the application itself.

One simple way to tune the thermal, optical and electrical properties, improving some effects and reducing some others non-desired, is by means of confinement [3–37]. Quenched random disorder on liquid crystals may induce smooth and/or drastic variations in their physical properties and, therefore, an exhaustive study of these systems is of great importance. Furthermore, liquid crystals in a device are strongly perturbed by confining interfaces, which means that under adequate confinement techniques, we are able to study how the properties will behave in actual micro- or nano-sized devices, and we may, indeed, be hitting two targets with one shot.

In this work, we are studying the influence on the thermal, dielectric and dielectric properties of calamitic liquid crystals affected by the random disorder induced by the dispersion of nano-sized γ-alumina particles, which are used for the first time for confining purposes. As shown recently [35], this kind of nanoparticles noticeably reduces the undesired ionic effects on liquid crystals, which goes in the good direction when the optimization of electrical properties is pursued. Furthermore, as we are about to demonstrate, they shift the phase transition temperatures down, in a very much drastic way as compared to other structures used for confinement purposes like, for example, Anopore membranes or Aerosil silica particles.

The material object of the present research is the butyloxybenzylidene octylaniline, hereafter refered as 4O.8, a smecto-nematogenic compound whose thermal properties in bulk [38,39], confined in silica aerogel matrices [12] and confined under the dispersion of Aerosil particles [13,14] have been analyzed earlier. This is, however, the first time their dielectric properties, in bulk as well as under confinement, are reported, as it was claimed to be non-polar so far [13,38]. We show in this work that the molecule has a small (compared to common cyanobiphenyls) but measurable dipolar transversal moment, which induces a negative dielectric anisotropy.

The results presented in this manuscript, all for bulk and confined samples, are addressed in two parts, one for each of the studied properties: (1) We start with the calorimetric data, in which we can observe the deep depression in phase transition temperatures and the change in the shape of the heat capacity (*C_p_*) peaks in the confined samples. Both effects are higher as the concentration of nanoparticles in the liquid crystal increases. A comparison between dispersions of γ-alumina and Aerosil nanoparticles is performed; (2) The behavior of the static and the complex dielectric permittivities, which allow us to interpret the molecular dynamics, is presented and discussed.

## 2. Results and Discussion

**Figure 1.**
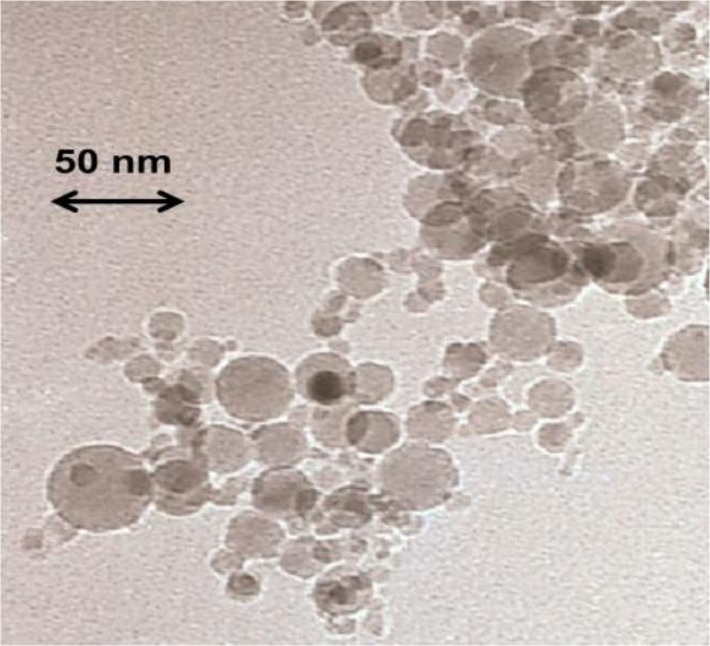
Transmition Electronic Microscopy picture of the γ-alumina nanoparticles.

In order to check the influence of the random confinement on the analysed physical properties of the 4O.8 compound, we have prepared ten different samples: one bulk 4O.8 sample (ρ_S_ = 0), seven more with different concentrations of γ-alumina nanoparticles (ρ_S_ = 0.01 g·cm^−3^, ρ_S_ = 0.05 g·cm^−3^, ρ_S_ = 0.13 g·cm^−3^, ρ_S_ = 0.17 g·cm^−3^, ρ_S_ = 0.19 g·cm^−3^, ρ_S_ = 0.23 g·cm^−3^ and ρ_S_ = 0.28 g·cm^−3^, where ρ_S_ accounts for density in grams of γ-alumina per cm^3^ of 4O.8) and the last two with hydrophilic Aerosil dispersions (ρ_S,AS_ = 0.05 g·cm^−3^ and ρ_S_,_AS_ = 0.23 g·cm^−3^). The main difference between the hydrophilic silica Aerosil and γ-alumina nanoparticles is that the formers are surrounded by OH radicals, whereas the latters are radical-free. In the case of hydrophilic Aerosil, when the weight concentration, ρ_S,AS_, of these particles exceeds a certain threshold (ρ_S,AS_~0.01 g·cm^−3^), the OH groups tend to get attached forming hydrogen bonds, which induce the formation of thixotropic gelly structures. Depending on the concentration, these gels can be soft (0.01 g·cm^−3^ < ρ_S,AS_ < 0.1 g·cm^−3^) or stiff (ρ_S,AS_ > 0.1 g·cm^−3^), and the quenching of the mesogenic material becomes more pronounced as the concentration increases. Alumina nanoparticles, however, are not able to form these hydrogen bonds, as they are not coated with any functional group. They are spherical and have diameter lengths ranging between 3 and 30 nm, as can be observed from the transmission electronic microscopy (TEM) photo in Figure 1, being the average diameter about 15 nm.

### 2.1. Thermal Analysis

The first issue to check is how the mesophases are affected by the dispersion of the nanoparticles. As mentioned above, 4O.8 is a smecto-nematogenic liquid crystal, and presents the following phase sequence on heating from room temperature: Crystal-Plastic Crystal-SmA-N-I. For the thermal analysis, we are focusing on the SmA-N and N-I phase transitions.

**Figure 2.**
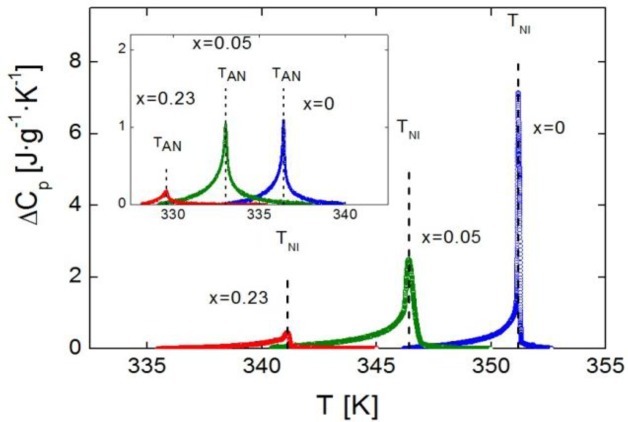
Heat capacity data as a function of temperature near the N-I phase transition (and near the SmA-N phase transition in the inset) of the system 4O.8 + γ-alumina, for the samples with concentrations ρ_S_ = 0 (bulk, blue circles), ρ_S_ = 0.05 g·cm^−3^ (green circles) and ρ_S_ = 0.23 g·cm^−3^ (red circles). Dashed lines indicate the N-I (and SmA-N in the inset) transition temperatures for each of the samples.

Figure 2 shows the excess heat capacity, Δ*C_p_*, behavior as a function of temperature, for bulk 4O.8 and two of the γ-alumina dispersed samples (ρ_S_ = 0.05 g·cm^−3^ and ρ_S_ = 0.23 g·cm^−3^), around the N-I phase transition. The SmA-N phase transition Δ*C_p_* peaks of the same three samples are displayed in the inset of Figure 2.

It can be observed that neither of the mesophases, SmA nor N, is suppressed by confinement. Furthermore, the more the concentration of nanoparticles, the more the transition temperatures are shifted down. It should be stressed that we are proving that the depression in transition temperatures is much more pronounced for confinement via γ-alumina than via Aerosil for the same concentration of nanoparticles, as shown in the values listed in Table 1. It can be seen how our data (in Aerosil) are quite in accordance with those from the work by Haga and Garland [13]. As it can be observed, and as a very interesting first result, moderate concentrations of γ-alumina nanoparticles are capable of shifting the SmA-N and N-I transition temperatures down to values around ten degrees below the bulk, and even more, while similar concentrations of Aerosil just make them decrease about two or three degrees.

**Figure 3.**
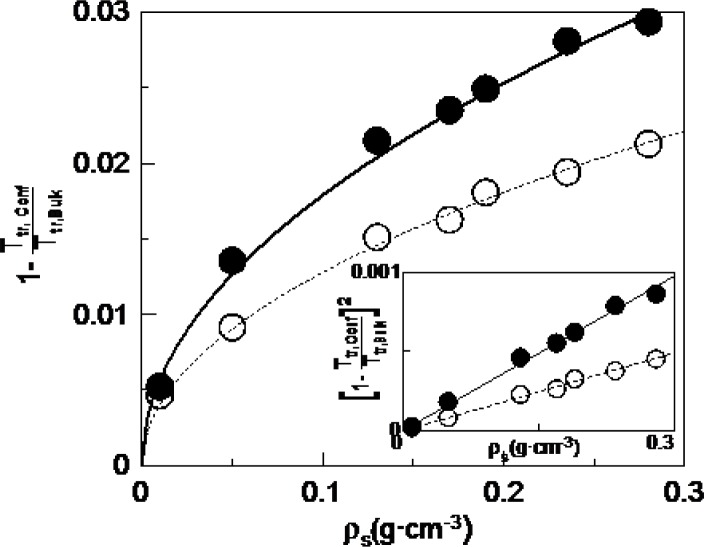
Absolute value of the depression of the transition temperatures divided by the bulk transition temperature for the N-I (full circles) and SmA-N (empty circles) phase transitions. Straight and dashed lines indicate the fittings to Equations (1a,b), respectively. The inset shows the corresponding linear relationships coming from squaring Equations (1a,b).

Figure 3 shows the dependence with concentration of the absolute value of the depression of the transition temperatures divided by the bulk transition temperature for the N-I (full circles) and SmA-N (empty circles) phase transitions. The experimental points for the studied concentrations can be fitted using the following equation (lines in Figure 3):

$$1-\frac{T_{NI,\rho_{s}}}{T_{NI,bulk}}=\sqrt{a_{NI}\rho_{s}}$$ (1a)

$$1-\frac{T_{AN,\rho_{\text{s}}}}{T_{AN,bulk}}=\sqrt{a_{AN}\rho_{s}}$$ (1b)

being *T*_*NI*,ρ*s*_ the N-I phase transition temperature at the considered concentration, ρ*_s_*; *T_NI,bulk_* the N-I phase transition temperature for the bulk, and *a_NI_* the fitting parameter for the N-I phase transition, while *T_AN,ρs_*, *T_AN,bulk_* and *a_AN_* are corresponding parameters for the SmA-N phase transition. In the inset of Figure 3, we can see how the corresponding linear fittings are quite good. This result suggest that the depression of transition temperatures with concentration follows a monotonous behavior. However, when trying to obtain similar relationships with the Aerosil data this is not possible, as they clearly present a change of behaviour when passing from the soft-gel regime to the stiff-gel regime (~0.1 g·cm^−3^). These results seem to indicate that with γ-alumina nanoparticles there is not such a distinction between soft and stiff-gel regimes as with Aerosil, which is in accordance with the fact that the former do not have functional groups in the surface.

Another important outcome of this research is that the values of the fitting parameter *a* are 3.2 × 10^−3^ cm^3^·g^−1^ for the N-I phase transition and 1.6 × 10^−3^ cm^3^·g^−1^ for the SmA-N phase transition. There is a factor of about 2 between both transitions. The fact that the N-I transition temperatures become more affected than those of the SmA-N one, may indicate that, even if both transitions are weakly first order in nature, the latter is more continuous (more directed to second order). What is really impressive is the ratio of about 2 between both *a_NI_* and *a_AN_* parameters, which shall be object of further research.

**Figure 4.**
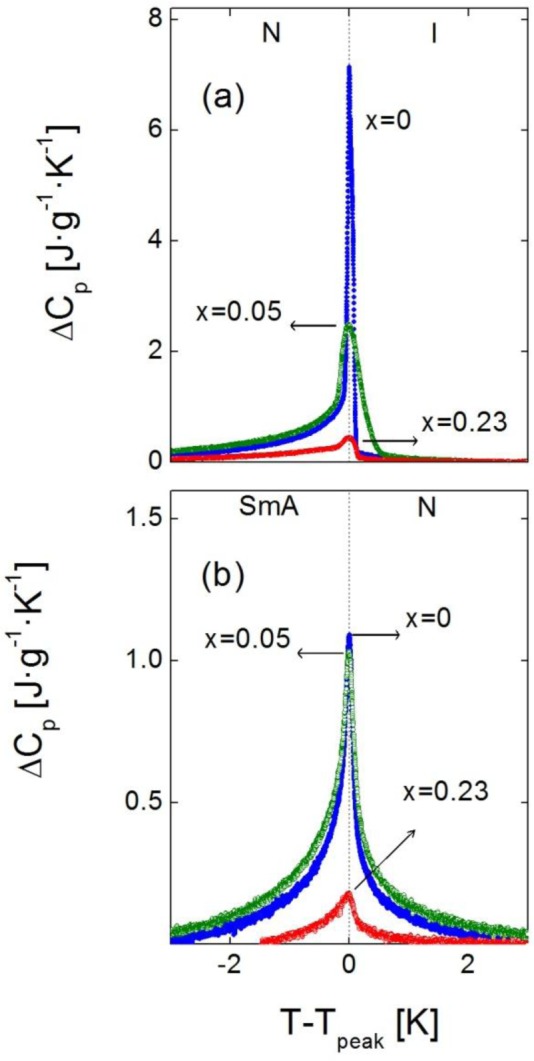
Comparison of the excess heat capacity peak shapes near the (**a**) N-I and (**b**) SmA-N phase transitions for different concentrations of the 4O.8 + γ-alumina system: ρ_S_ = 0 (blue circles), ρ_S_ = 0.05 g·cm^−3^ (green circles) and ρ_S_ = 0.23 g·cm^−3^ (red circles). Dashed lines indicate the phase transitions.

In addition to the change in transition temperatures, the influence of the confinement is remarkable in the shape and height of the *C_p_* peaks in both the SmA-N and N-I phase transitions. As the concentration of nanoparticles increases, the *C_p_* peaks become broader, rounded and shorter in height. This effect, that can be observed for the samples with ρs = 0, 0.05 and 0.23 g·cm^−3^ in Figure 4a (N-I phase transition) and 4b (SmA-N phase transition), is qualitatively similar to what occurs in other types of liquid crystals under confinement [4,10,11,13,16,24,28,31,33]. A quantitative measure of the broadening of the peaks is done by means of their full width at half maximum (FWHM). The values of the FWHM in the N-I *C_p_* peaks increase from 0.1 K in the bulk sample to ~0.3 K in the ρ_S_ = 0.01–0.19 g·cm^−3^ samples, arriving up to ~0.5 K in the ρ_S_ = 0.23 g·cm^−3^, 0.28 g·cm^−3^ samples. For the SmA-N peaks, FWHM goes from 0.2 K in the bulk to ~0.5 K in the confined samples. These values are similar to those corresponding to the dispersion of Aerosil nanoparticles in the same liquid crystal for small concentrations (soft-gel regime), as confirmed by the ρ_S,AS_ = 0.05 g·cm^−3^ sample, where the FWHM is 0.3 K for the N-I peak and 0.5 K for the SmA-N one. Nevertheless, such values are much smaller than those for high concentrations (stiff-gel regime) of Aerosil in 4O.8, as for the ρ_S,AS_ = 0.23 g·cm^−3^ sample, the FWHM of the N-I and SmA-N peaks are 0.8 K and 1.4 K, respectively. This confirms the fact that, at least, the γ-alumina particles do not form stiff-gels, as the Aerosil particles do. But the FWHM values are close to those samples comparable in concentration with Aerosil soft-gel regime ones, which means that FWHM measurements do not rule out the formation of soft-gels with γ-alumina nanoparticles. The question is, therefore, if γ-alumina nanoparticles can form any kind of soft-gel or similar, even if they cannot form hydrogen bonding networks.

Regarding the experimental results about the drastic shifting down of transition temperatures together to the lower (compared to Aerosil) effect in the *C_p_* peak-shapes for the confinement by means of γ-alumina nanoparticles, we may propose a possible explanation: Might be that when Aerosil particles form thixotropic gel structures small “islands” of liquid crystal are embedded inside the gel hollows. Such structures are much more uniform and homogeneous than the combination of the liquid crystal with dispersing γ-alumina particles, which might just act like “simple” impurities. The homogeneity of the LC + Aerosil structures could induce local anisotropic disordering, implying a drastic change with respect to the nature of the bulk liquid crystal, which is reflected in a marked variation in the nature of the phase transitions and, so, in the shape of the *C_p_* peaks at the transitions. In the other hand, the LC + γ-alumina system simply lowers the transition temperatures but, at the same time, it just slightly changes the transitions’ nature. The γ-alumina nanoparticles do not considerably alter the liquid crystalline structure, but they do introduce an isotropic disorder that just shifts the mesophases down in temperature. Such a distinction between gel structures and LC + impurities becomes clearer when particle concentration is high, which could explain why at low concentrations (soft-gel regime for Aerosil) the *C_p_* peak shapes in both N-I and SmA-N phase transitions are similar for both kinds of confinements. Anyhow, this remains an open question and more experiments must be performed in order to clarify and understand the behaviour of such confinements. Following this argument, we are now carrying on studies with γ-alumina nanoparticles dispersed in other kinds of liquid crystals [40].

Besides, the broadening of the *C_p_* peaks means that the weakly first order phase transitions become even weaker with confinement and, eventually, might be driven to second order in nature [28,33]. Further exhaustive studies should be done in order to determine the critical behavior of these phase transitions, and see how it evolves with changing the concentration of nanoparticles, but such an analysis is beyond the scope of the present work.

### 2.2. Dielectric Analysis

First of all, it must be said that, in the dielectric measurements, the depressions in phase transition temperatures are not as pronounced as in the calorimetric ones. The difference in the samples themselves as well as the added interaction of the applied electric field, drastically change the transition temperatures behaviour with respect to the high resolution calorimetry experiments. Anyway, in this section we will just focus on the dielectric properties of the bulk 4O.8 and the influence of confinement in these properties alone. It should be stressed that such kind of studies are being performed for the first time in bulk 4O.8.

**Scheme 1.**
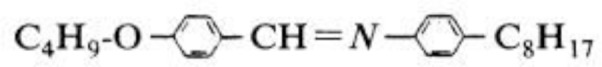
Butyloxybenzylidene octylaniline (4O.8) molecule.

Several studies have claimed for the non-polarity of 4O.8 [13,38]. Nevertheless, the 4O.8 molecule has a mainly transverse dipole moment due to the imine group, as can be seen in Scheme 1.

There is another transverse dipole due to the oxygen atom, that can be ruled out, in comparison with that of the imine group. Therefore, 4O.8 should present a negative dielectric anisotropy due to the mainly transverse dipole moment. In order to measure this response, measurements of the static and the complex dynamic dielectric permittivitty in the I, N and SmA phases have been performed, for bulk 4O.8 and for confined samples.

#### 2.2.1. Static Dielectric Permittivity

**Figure 5.**
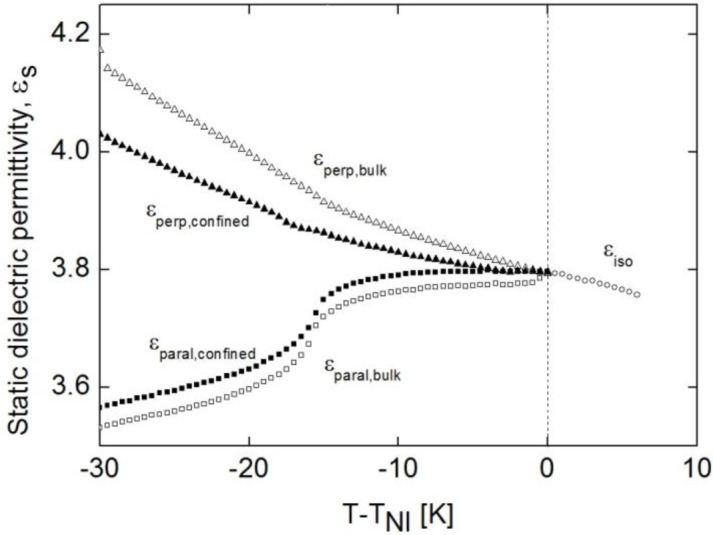
Static dielectric permittivity *vs*. temperature for the ρ_S_ = 0 (bulk) and ρ_S_ = 0.28 g·cm^−3^ samples of the 4O.8 + γ-alumina system: Empty circles correspond to the isotropic phase; triangles correspond to the perpendicular component of the static dielectric permittivity in the mesophases, empty triangles for the ρ_S_ = 0 sample and full triangles for the ρ_S_ = 0.28 g·cm^−3^ sample; squares represent the parallel component of the static dielectric permittivity in the mesophases, empty squares for ρ_S_ = 0 and full squares for ρ_S_ = 0.28 g·cm^−3^. The dashed line marks the N-I phase transition.

As the perpendicular component of the static dielectric permittivity, ε_⊥_, is higher than the parallel component, ε_||_, the dielectric anisotropy of the molecules is negative (Δε = ε_||_ − ε_⊥_). This can be checked in Figure 5, were both components are presented *versus* T-T_NI_, for two of the bulk, along with the static dielectric permittivity value in the isotropic phase. These measurements have been performed at 10 kHz, a frequency high enough to rule out the conductivity and ionic contributions and, at the same time, lower than that of the orientational contributions. The behavior of ε_║_ and ε_⊥_, for bulk as well as for confined samples, is the typical of calamitic liquid crystals with negative dielectric anisotropy [41]. Comparing to the bulk, the confined samples have a smaller dielectric anisotropy (in absolute value) in the N mesophase, which is nearly zero close to the N-I phase transition.

#### 2.2.2. Molecular Dynamics: Relaxation Modes

##### Bulk Sample

**Figure 6.**
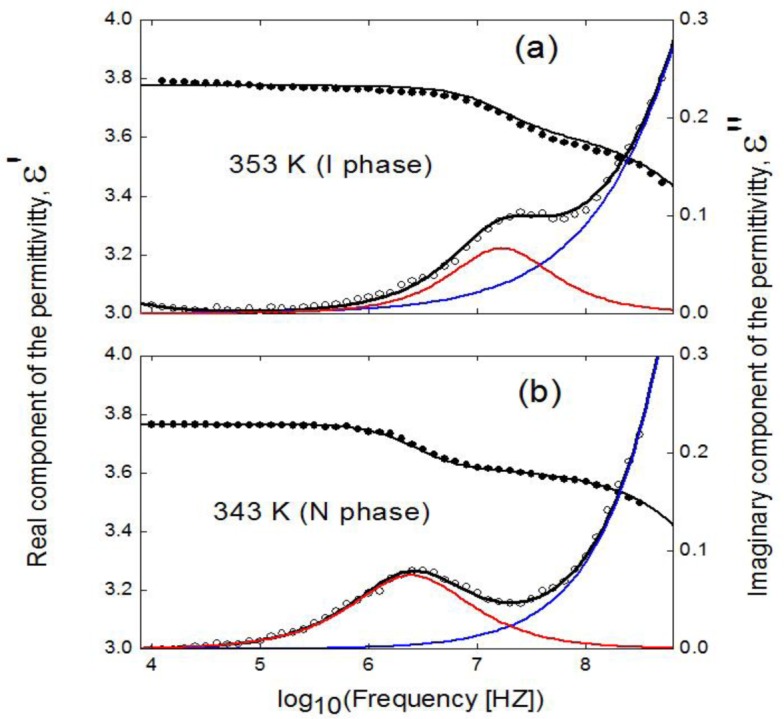
Frequency dependence of the real (full circles) and imaginary (empty circles) parts of the complex dielectric permittivity of the bulk 4O.8 (**a**) in the isotropic phase (*T* = 353 K) and (**b**) in the nematic phase (*T =* 343 K) in a likely-planar alignment. Black solid lines are fittings according to Equation (2). Red and blue lines represent deconvolution into the imaginary parts of the ω_1_ and ω_2_ modes, respectively. For simplicity, the direct current conductivity contribution (σ_dc_) is not drawn, but is considered in the fitting procedure.

Figure 6 shows both the real and imaginary parts of the complex dielectric permittivitty for bulk 4O.8, in both the I and N phases, the latter in a likely-planar alignment (molecules mainly parallel to the cell surfaces and perpendicular to the probing electric field). Spontaneously, the sample adopts a mixed alignment when placed into the metallic cell. When a bias DC field of 35 V is applied, the compound adopts a likely-planar alignment, as the material has a negative dielectric anisotropy. Any attempts to get the homeotropic alignment (molecules perpendicular to the cell surfaces and parallel to the probing electric field) was unfruitful. It can be observed the existence of two relaxation processes in both the I and N phases, the one at higher frequencies (around 1–10 GHz) with higher amplitude (about 3–5 times, depending on temperature) than the one at lower frequencies (1–10 MHz). Such a ratio in the strength of the modes is coherent with the fact that the dipolar moment is almost transverse (negative dielectric anisotropy). The low frequency mode (denoted as ω_1_, from now on [42]) is due to the end-over-end rotations of the molecules around their short axis and the high frequency one (denoted as ω_2_, from now on [42]) is due to the coupling of two types of molecular motions: rotations around their long axes and precessions around the nematic director. When cooling down to the SmA phase from the N, the behavior of the modes does not change significantly, being the spectra similar in both mesophases.

The experimental results of the complex dielectric permittivity, ε(ω), have been fitted through the empirical function:

$$\varepsilon (\omega )=\underset{k}{\sum }\frac{\Delta \varepsilon_{k}}{{\left[1+{\left(i\omega \tau_{k}\right)}^{\alpha_{k}}\right]}^{\beta_{k}}}+\varepsilon_{\infty }-i\frac{\sigma_{DC}}{\omega \varepsilon_{0}}$$ (2)

where the summation is extended over the two relaxation modes, and each one is fitted according to the Havriliak-Negami function; *k* = 1, 2 are the different relaxation processs; Δε*_k_* and *τ_k_* are the dielectric strength and a relaxation time related to the frequency of maximum dielectric loss, respectively, of each relaxation mode; *α_k_* and *β_k_* are the parameters that describe the shape (width and symmetry) of the relaxation spectra; ε_∞_ is the dielectric permittivity at high frequencies (but lower than those corresponding to atomic and electronic resonance phenomena) and σ*_DC_* is the electric conductivity. According to the fits, ω_1_ is a Debye relaxation mode (α = β = 1). The high frequency mode (ω_2_) lays in the brink of the frequency window and, therefore, the values coming from the fitting of such a mode must be taken with extreme caution. Although in Figure 6 the ω_2_ mode is in the brink of the frequency window, it can be characterized at sufficiently low temperatures. This way, we have been able to fit the parameters of such a mode through Equation (2) at high temperatures, coming from lower temperatures and following a coherent behavior in their temperature dependences. Anyway, these fitting values must be taken with extreme caution, and we can temptativously say that ω_2_ is a Cole-Davidson mode (α = 1 ≠ β), with β ranging from 0.8 at high temperatures to 0.9 near the SmA-N phase transition.

**Figure 7.**
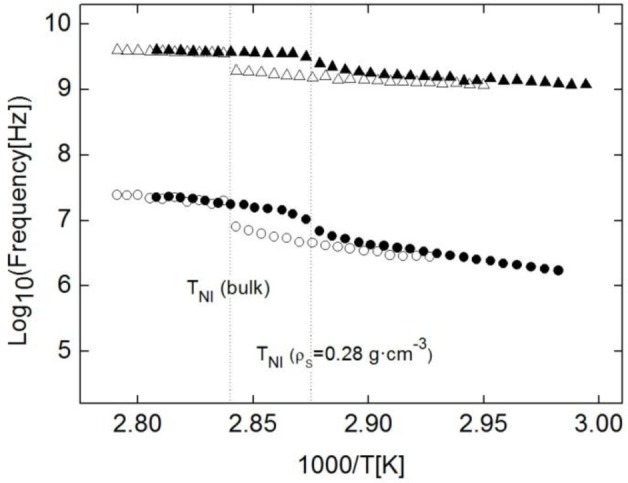
Arrhenius plot of the relaxation frequencies of the ω_1_ (circles) and ω_2_ (triangles) modes for the bulk 4O.8 (empty symbols) and ρ_S_ = 0.28 g·cm^−3^ (full symbols) samples of the 4O.8 + γ-alumina system. Dashed lines indicate the N-I phase transitions for both samples.

The frequency dependence on temperature for both modes can be seen in the Arrhenius plot in Figure 7 (empty symbols). The presence of two relaxation modes in the isotropic phase is typical of materials with an anisotropic rotational diffusion tensor. The behavior of both modes is as expected for these kinds of molecular reorientations. The frequency jump for the ω_2_ mode at the N-I phase transition is very smooth, and the activation energy of this relaxation is quite low, as expected for this kind of reorientations. On the other hand, ω_1_ jumps down clearly in frequency when going from I to N and its activation energy is higher than that corresponding to the ω_2_ mode. The validity of the fitting of ω_2_, although limited, can be taken as optimal as its tendency is typical of these kind of relaxation modes in rod-like molecules [25,26,28,43], as can be seen from the Arrenius plot in Figure 7.

##### Confined Samples

The results of the confined samples (4O.8 + γ-alumina as well as 4O.8 + Aerosil) do not show noticeable differences with respect to the bulk ones, nor in amplitudes, neither in frequencies of the relaxation modes, but only the fact that they spontaneously adopt a planar alignment without a DC bias. In some cases, the spontaneous arrangement of calamitics in untreated metallic cells tends to be planar or nearly planar [43,44], though this is not always true and, sometimes, the cell surfaces must be treated with some surfactant in order to achieve the desired planar alignment of the molecules [25]. Fluidity of the nematic phase induces an average disorder of the molecules and, therefore, of the global nematic director, which translates in an inhomogeneous alignment of the sample, as is the case of the bulk 4O.8 sample. Nevertheless, the introduction of nanoparticles (γ-alumina as well as Aerosil) produces a decrease in fluidity and. Intriguingly, even if it induces random disorder, when combined with the applied electric field, it seems to increase the orientational order in a molecular planar alignment. Hence, the inhomegeneity within the metallic cell diminishes and the molecular arrangement is more planar-like. If this were the case, it should induce the homeotropic molecular alignment in samples of liquid crystals with positive dielectric anisotropy confined by the dispersion of hydrophilic (polar) nanoparticles. We are currently trying to study this phenomenon with some liquid crystals with longitudinal net dipole moments.

With respect to the relaxation modes, the behavior is similar to that of the bulk sample, with the same ω_1_ and ω_2_ modes. As in the bulk, ω_1_ is Debye-like and ω_2_ is, temptatively, Cole-Davidson, with β ranging from 0.6 to 0.8 as temperature decreases. Full symbols in Figure 7 show the frequency dependence on temperature of both modes for the ρ_S_ = 0.28 g·cm^−3^ sample. As it can be observed from this figure, the values are similar to those from the bulk sample, and this result can be reproduced for each of the studied samples. Such results mean that not only qualitatively, but also quantitatively, the dielectric behaviour of the planar samples is the same, irrespective of the given concentration (from ρ_S_ = 0 to ρ_S_ = 0.28 g·cm^−3^) and the nature (γ-alumina or Aerosil) of nanoparticles. As can be deduced from these results, fluidity differences between bulk and confined samples do not influence at all molecular reorientations.

## 3. Experimental Section

### 3.1. Preparation of the Materials

The pure 4O.8 compound was synthesized and purified at the Institute of Chemistry, Military University of Technology, Warsaw, Poland. Its purity was stated to be 99.6% and no further purification was made.

The alumina nanoparticles, in the γ-phase, were commercially obtained from Tecnan (Los Arcos, Spain) and their purity was claimed to be 99.995%. The density of the nanoparticles is about 3.65 g·cm^−3^ and they have a surface area of about 110 m^2^·g^−1^. The particles, which are not coated with any functional group, are spherical and have diameter lengths ranging between 3 and 30 nm, as can be observed from the transmission electronic microscopy (TEM) photo in Figure 1, being the average diameter about 15 nm.

The silica spherical particles, of the type Aerosil 300, were commercially obtained by Degussa (Frankfurt, Germany). They are hydrophilic, with OH radicals in the surface and an average diameter of 7 nm. The quoted values of density and surface area are 2.6 g·cm^−3^ and 300 m^2^·g^−1^, respectively. TEM measurements confirm that, as the γ-alumina particles, Aerosil particles present a polydispersive nature.

The dispersion of the nanoparticles in the pure compound was made mechanically, in an ultrasound bath in the isotropic phase (I) at temperatures slightly above the N-I transition temperature for the pure compound. The homogeneity of the mixtures was stated by the high resolution heat capacity measurements and the methodology of preparation was validated by comparison with bibliographical data for the Aerosil dispersions of the same liquid crystal [13].

### 3.2. Transmission Electronic Microscopy

A field transmission electronic microscope JEOL JSM-7001F was used. The sample was metallized with Pt-Pd, and the photo of Figure 1 was obtained at 200 K and 120 keV.

### 3.3. Heat Capacity Measurements

Static heat capacity data at constant pressure were obtained through the Modulated Differential Scanning Calorimetry (MDSC) technique via a commercial TA instruments Q-2000 (New Castle, DE, USA), for which extensive details can be found elsewhere [45]. Similar to an AC calorimeter, the MDSC technique, in addition to heat capacity data, simultaneously provides phase shift data (δ) that allow determining the phase coexistence region in weakly first order transitions. The experimental conditions were adjusted in such a way that the phase delay (δ) between the modulated heat flow (the response to the perturbation) and the induced temperature oscillations (perturbation) was nearly zero out of the phase transition, and the imaginary part of the complex heat capacity data vanished.

Typically, measurements were performed on heating from the crystal phase up to the isotropic phase; the temperature rate was 0.01 K·min^−1^ with a modulation temperature amplitude (temperature oscillations) of ±0.07 K and a period of 23 s.

### 3.4. Dielectric Measurements

Measurements of the static dielectric permittivity, ε*_S_*, were performed using the HP 4192A impedance analyzer. We have used two different kind of square glass cells, depending on the desired alignment of the molecules with respect to the probing electric field. For measuring the parallel component of the static dielectric permittivity homeotropic alignment (molecules perpendicular to the cell surfaces) is required. Otherwise, for the study of the perpendicular component of the static dielectric permittivity, molecules must be in planar alignment (parallel to the cell surfaces). Both types of cells are from Instec (Boulder, CO, US), with an electrode surface of 1 cm^2^ and thicknesses of 9 μm (homeotropic cells) and 8 μm (planar cells). The temperature control was made by a Linkam THMSG-600 hot stage and a Linkam TMS-94 temperature controller (Waterfield, Surrey, UK).

Measurements of the complex dielectric permittivitty were performed by means of two different equipments, depending on the analyzed frequency range. For radio frequencies (1 MHz to 1.8 GHz) the measurements were made by means of a HP 4291A impedance analyzer. For audio frequencies (1 kHz to 10 MHz) the HP 4192A impedance analyzer was used. The cell consists of two gold-plated brass electrodes (diameter 5 mm) separated by silica spacers, making a plane capacitor of about 50 μm thick. A modified HP 16091A coaxial test fixture was used as the sample holder. It was held in a cryostat made by Novocontrol (Hundsangen, Germany), and both temperature and dielectric measurements were computer controlled. Additional details of the experimental technique can be found somewhere else [43,45,46]. Dielectric measurements were performed on cooling with stabilization at different temperature steps and a temperature control on the order of 20 mK.

## 4. Conclusions

In the present work, we have presented a high resolution calorimetric and dielectric study both in 4O.8 bulk and in confined samples of γ-alumina nanoparticles dispersed in 4O.8.

The dispersion of γ-alumina nanoparticles in 4O.8 shifts downwards both N-I and SmA-N phase transition temperatures. The higher the concentration of nanoparticles, the lower the transition temperatures are with respect to the bulk. This effect is typical of confined systems, but in this case the decrease of the phase transition temperatures is much more pronounced than with any other type of confinement studied so far. For comparable concentrations of nanoparticles (γ-alumina and Aerosil), the *T*_NI_ shifts down three times more (low concentrations) and four times more (high concentrations) in the case of γ-alumina than in the case of Aerosil. Regarding the *T*_AN_, the shifting down from the bulk is about twice more (low concentrations) and three times more (high concentrations).

An important experimental result to compare confinement by means of γ-alumina or Aerosil is the possibility of fitting data with γ-alumina to Equations (1a,b), which cannot be done for Aerosil data, in which there is a clear change of behavior. This suggests that γ-alumina nanoparticles, unlike Aerosil, do not present two different regimes, *i.e*., a soft-gel regime below concentrations of about 0.1 g·cm^−3^ and a stiff-gel regime above. The relationship of these fittings between both N-I and SmA-N phase transitions is really intriguing as there is a correspondence of about 2:1 between the *a_NI_* and the *a_AN_* fitting parameters. Additional studies should be undertaken.

The other thermal effect caused by the confinement is the change in the shape and height of the heat capacity peaks at phase transitions. In the studied case, this change of shape (reflected as a brodening) is not as noticeable as for the confinement by means of Aerosil. The values of the FWHM of the *C_p_* peaks of the 4O.8 + γ-alumina are similar than those of Aerosil dispersions for the so-called soft-gel regime (ρ_S_ < 0.1 g·cm^−3^), but are much smaller for the stiff-gel regime (ρ_S_ > 0.1 g·cm^−3^). In summary, γ-alumina nanoparticles introduces a disorder in the compound, *i.e*., it favours the more disordered phases with respect to the more ordered ones, as observed by a notable lowering of both N-I and SmA-N transition temperatures. Althoug this also happens by means of Aerosil dispersion, with the γ-alumina it is much more pronounced. At the same time, confinement by means of γ-alumina does not affect the nature of the phase transitions as much as Aerosil particles do for high concentrations (ρ_S_ > 0.1 g·cm^−3^). This seems to indicate that the γ-alumina nanoparticles, at least do not form stiff-gels, like the Aerosil does.

The most noticeable change in the dielectric results consequence of the confinement is that the dielectric anisotropy is less negative than in the bulk. The dynamic dielectric properties are similar for bulk and confined samples, presenting two relaxation modes (reorientations around short and long molecular axes) whose frequencies do not depend on nanoparticles concentration whatsoever. There is, nevertheless, one interesting influence of the γ-alumina (and Aerosil also) particles, which is the induced ordering of the molecules facilitating planar configurations in metallic samples, when bulk 4O.8 adopts an inhomogenous fashion.

Finally, we would like to say that the 4O.8 + γ-alumina system could be an optimal candidate for electro-optical applications, for the following reasons: (1) the nematic range can get closer to room temperature with concentration of nanoparticles and, so, more accessible for device applications; (2) dielectric properties do not suffer significant changes due to confinement, which is interesting in the meaning that molecular dynamics is not slowed down and electro-optical response is, therefore, fast enough for switching applications; (3) it seems that γ-alumina nanoparticles improve the molecular orientational ordering with the dipolar moment parallel to the applied electric field.

## Figures and Tables

**Table 1.** N-I and SmA-N phase transition temperatures and nematic range (NR) for the studied samples.

ρ_S_ (g·cm^−3^)	T_NI_ (K)	T_AN_ (K)	NR (K)
0 (bulk 4O.8)	351.19	336.42	14.77
0.01	349.35 (−1.84)	334.86 (−1.56)	14.49
0.05	346.43 (−4.76)	333.34 (−3.08)	13.09
0.13	343.64 (−7.55)	331.34 (−5.08)	12.30
0.17	342.93 (−8.26)	330.95 (−5.47)	11.98
0.19	342.44 (−8.75)	330.34 (−6.08)	12.10
0.23	341.32 (−9.87)	329.86 (−6.53)	11.46
0.28	34O.88 (−10.31)	329.26 (−7.16)	11.62
ρ_S,AS_ = 0.05	349.54 (−1.65)	334.64 (−1.78)	14.90
ρ_S,AS_ = 0.23	348.48 (−2.71)	333.89 (−2.53)	14.59
